# Histidine-Tryptophan-Ketoglutarate Cardioplegia Yields Different Results in Aortic Valve Surgery Depending on Patient Gender: A Pilot Study

**DOI:** 10.7759/cureus.67372

**Published:** 2024-08-21

**Authors:** Claudiu Ghiragosian, Marius Harpa, Alexandra Puscas, Radu Balau, Hussam Al-Hussein, Simina-Elena Ghiragosian-Rusu, Calin Avram, Dragos-Florin Baba, Radu Neagoe, Horatiu Suciu

**Affiliations:** 1 Cardiac Surgery, Department of Surgery IV, George Emil Palade University of Medicine, Pharmacy, Science, and Technology of Târgu Mureș, Târgu Mureș, ROU; 2 Cardiovascular Surgery, Department of Surgery IV, George Emil Palade University of Medicine, Pharmacy, Science, and Technology of Târgu Mureș, Târgu Mureș, ROU; 3 Cardiovascular Surgery, Department of Anatomy-Embryology M1, George Emil Palade University of Medicine, Pharmacy, Science, and Technology of Târgu Mureș, Târgu Mureș, ROU; 4 Pediatric Cardiology, Department of Pediatrics III, George Emil Palade University of Medicine, Pharmacy, Science, and Technology of Târgu Mureș, Târgu Mureș, ROU; 5 Department of Medical Informatics and Biostatistics, George Emil Palade University of Medicine, Pharmacy, Science, and Technology of Târgu Mureș, Târgu Mureș, ROU; 6 Cardiology, Department of Cell and Molecular Biology, George Emil Palade University of Medicine, Pharmacy, Science, and Technology of Târgu Mureș, Târgu Mureș, ROU; 7 2nd Department of Surgery, George Emil Palade University of Medicine, Pharmacy, Science, and Technology of Târgu Mureș, Târgu Mureș, ROU

**Keywords:** histidine-tryptophan-ketoglutarate cardioplegia, cardioprotection, postoperative mortality, aortic valve replacement, bretschneider cardioplegia

## Abstract

Background: Histidine-tryptophan-ketoglutarate (HTK) cardioplegia induces cardiac arrest through membrane hyperpolarization. Aortic valve pathology leads to pathophysiological changes in left ventricular vascularization that may prevent adequate cardioplegic distribution. The objective of the study was to ascertain whether the use of Bretschneider cardioplegia in aortic valve surgery yields different outcomes for male and female patients.

Methodology: Our study compares the perioperative data of 300 adult patients who underwent aortic valve replacement between June 2023 and June 2024 at the Emergency Cardiac Disease and Transplant Institute, Tîrgu Mures, Romania. Concomitant procedures, age under 18 years, retrograde or combined cardioplegia, and emergency surgery were excluded. The main outcome was operative mortality, and secondary outcomes were postoperative complications and paraclinical data such as ejection fraction and cardiac enzymes.

Results: Male patients comprised 190 (62%) of the sample. The most common age group was 61-70 years in both groups. The mortality rate was 6 (5.4%) for women compared to 9 (4.7%) for men. Preoperative left ventricular ejection fraction was the primary covariate determining 30-day postoperative mortality. Left ventricular ejection fraction decreased by 2.2% in men and 1.1% in women 30 days after surgery.

Conclusions: The myocardial adaptation to aortic valve pathology exhibits gender-specific differences. However, the utilization of HTK cardioplegia obviates this disparity.

## Introduction

Aortic valve surgery is the most common valve procedure, accounting for 46.6% of all valve procedures [1]. Patients diagnosed with aortic valvulopathy are predominantly male (61.8% vs. 38.2% female) in the 5-6th decade of life [2-4]. Many factors can contribute to aortic valve dysfunction, including systemic arterial hypertension, dyslipidemia, diabetes, smoking, endocarditis, aortitis, congenital lesions (e.g., bicuspid aortic valve), connective tissue disorders (e.g., osteogenesis imperfecta, Marfan syndrome, Ehlers-Danlos syndrome) and trauma. The group of aortic valve diseases is dominated by aortic stenosis. It is estimated that between 2% and 9% of the general population over the age of 65 will develop this type of valvular disease, with a variable degree of transvalvular gradient. As a result of pressure overload (aortic stenosis) or volume overload (aortic regurgitation), the left ventricle (LV) adapts to maintain ejection fraction (EF). These mechanisms, which maintain an adequate hemodynamic status for a limited period, are detrimental to the left ventricular muscle mass itself. There is a sex difference in LV adaptation to pressure/volume overload. Women develop concentric remodeling, a higher degree of diastolic dysfunction, and a higher RWT (relative wall thickness) than men (who have eccentric hypertrophy and therefore increased VS dilatation) [5]. These changes in the coronary microcirculation are more pronounced in concentric hypertrophy (i.e. women), making the heart more vulnerable to the adverse effects of cardiopulmonary bypass [6]. A further significant morphopathological alteration pertains to the coronary microvascularization. In a hypertrophic LV, where there is an increase in the number and size of myocytes, there is a concomitant decrease in the number of capillary/myocardial cells, which is referred to as a reduction in capillary density. The reduction in microvascular density is directly proportional to the extent of cellular hypertrophy. Moreover, the thickening of the medial layer of the intramyocardial vessels and intimal hyperplasia (an increased wall/lumen ratio) result in an increase in coronary microvascular resistance. Coronary microvascular dysfunction is present in both the subepicardial and subendocardial layers. These pathological changes can present a challenge during cardioplegic administration in a hypertrophic LV, as the adequacy of cardioplegic distribution may be hindered. Some concerns have been expressed regarding the administration of Bretschneider cardioplegia at low pressure, particularly in the presence of a hyper-trophic LV or severe coronary lesions.

Histidine-tryptophan-ketoglutarate (HTK) or Bretschneider cardioplegia is an intracellular-type cardioplegia that induces diastolic cardiac arrest via membrane hyperpolarization, which occurs as a result of the low-- pressure infusion of a high-volume crystalloid-based formula.

In light of the observed changes in myocardial microvascularization and the documented differences in LV adaptability between genders, this study aims to compare the postoperative outcome between the sexes after aortic valve surgery when a low-pressure infusion of Bretschneider solution is used. The objective is to ascertain whether these modifications affect the postoperative outcome. Additionally, the interdependence between specific postoperative covariates has been investigated. To the best of our knowledge, few studies regarding postoperative outcomes after aortic valve surgery have analyzed these data according to patients' gender.

## Materials and methods

This is a retrospective pilot study, the aim of which is to review the clinical aspects, operative characteristics, and outcomes of patients who underwent aortic valve replacement (AVR) under the protection of HTK cardioplegia between June 2023 and June 2024 at the Emergency Institute for Cardiovascular Diseases and Transplantation, Tîrgu Mures, Romania.

The study included patients who had undergone elective surgery for adult AVR (regardless of prosthesis type), due to severe aortic stenosis or insufficiency, whether congenital or acquired. The exclusion criteria were as follows: concomitant procedures, age under 18 years, retrograde or combined cardioplegia, and emergency surgery. All study procedures were conducted in accordance with the ethical standards set forth in the Declaration of Helsinki, and the institutional review board approved the hospital's analysis protocol (protocol code 3240, April 15, 2024).

Data collection involved the sampling of blood from all patients at three key time points: one day before surgery (T1), at the time of ICU admission (T2), and on the second day after surgery (T3). Moreover, myocardial injury was quantified by analyzing the growth patterns of specific cardiac enzymes on postoperative day 1 in comparison to the value observed on the day before surgery. The enzymes in question were high-sensitivity troponin I (hs-cTnT) and creatine kinase MB isoenzyme (CK-MB). Transthoracic echocardiographic (TTE) measurements were conducted at the time of admission to the cardiac surgery ward, 24 hours later, and subsequently at various intervals, dependent on the patient’s evolution. Transesophageal echocardiography (TEE) was conducted before and following the resumption of cardiopulmonary bypass. Furthermore, left ventricular ejection fraction (LVEF) was calculated using TTE seven days postoperatively and recorded. The echocardiographic data included in the present study were documented by a single cardiologist.

The cardioplegia solution, developed by Bretschneider, was employed as a crystalloid substitute with antegrade administration at a temperature of 4 °C, as previously described by Ghiragosian et al., with re-administration at 90 minutes during aortic cross-clamping [7]. The cardioplegia solution was infused according to the manufacturer's recommendations.

The primary objective of this study is to determine the operative mortality (OM) rate. Secondary objectives include a comparison of postoperative ejection fraction with the preoperative state, as well as an evaluation of postoperative complications, including myocardial infarction (MI), arrhythmic rhythm onset, neurological dysfunctions, enzymatic dynamics, and incidence of postoperative bleeding.

Definitions

operative death is defined as any death occurring within 30 days of the surgical intervention or up to the time of discharge. Neurological dysfunction is defined as a symptomatic transient psychotic syndrome, stroke, or coma that is non-pharmacologically induced. All cases of new-onset postoperative atrial fibrillation were included in the study. The present study focused exclusively on ventricular arrhythmias that had developed after the patient's admission to the intensive care unit. In the present study, the authors specify diuresis (mean mL/day) as recorded throughout the hospitalization period in the intensive care unit. Additionally, the cases of re-exploration for hemostasis were recorded, which refers to repeated intervention for cardiac tamponade or bleeding from the time of ICU admission until discharge, except for hemorrhagic events resulting from subsequent medical procedures, such as the suppression of chest tubes or temporary pacemaker wires.

Statistical analysis

The data were classified as nominal or quantitative variables. The frequencies of nominal variables were employed to characterize them. The normality of the distribution of quantitative variables was tested using the Kolmogorov-Smirnov test and characterized by median and percentiles (25%-75%) or by mean and standard deviation (SD), as appropriate. The chi-square test was used to determine if there were associations between the nominal variables. The significant differences for the quantitative variables were identified using either the t-test or the Mann-Whitney test depending on the result of the normality of the data. We used logistic regressions to identify associations of postoperative cardiac enzymes dependent on specific covariates. The level of statistical significance was set at *P* < 0.05.

## Results

A total of 300 cases were identified through the research process. Demographic analysis revealed that a greater proportion of male patients (*n *= 190, 62.9%) underwent isolated AVR. Concerning age distribution, the cohort of patients aged 61 to 70 is the most prevalent in the female group (*n *= 56, 50.91%), as is the male group (*n *= 82, 43.16%). The proportion of elderly patients (over 70 years old) in both the male and female groups remains consistent (Table 1).

**Table 1:** Age distribution among the groups. Chi-square test; data were represented as *n* (%); *P* < 0.05 (statistically significant).

Age group (years)	Male (*n *= 190), *n* (%)	Female (*n *= 110), *n* (%)	*P*-value
21-30	0 (0.00%)	1 (0.91%)	0.395
31-40	2 (1.05%)	0 (0.00%)	
41-50	15 (7.89%)	7 (6.36%)	
51-60	42 (22.11%)	18 (16.36%)	
61-70	82 (43.16%)	56 (50.91%)	
>70	49 (25.79%)	28 (25.46%)	

Concerning the total intervention, bypass, and ischemic time of the surgical procedures, there was no notable difference between the two groups (Table 2). The quantity of ultrafiltration is approximately equivalent between the two groups. The number of defibrillations is presented as a measure of intrapericardial external electric shocks and the number of cases in which these procedures were necessary was similar in both groups. A comparison of the mean EF between the two groups on the day preceding surgery and seven days following surgery revealed higher values for women both preoperatively and postoperatively.

**Table 2:** Perioperative variables. *Unpaired t-test. **Mann-Whitney test. *P* < 0.05 statistically significant.

Variables	Male (*n* = 190)	Female (*n* = 110)	*P*-value
Total intervention time (min.) (Mean ± SD)	363.91 ± 86.39	367.82 ± 79.84	0.69*
Bypass time (min.) (Mean ± SD)	105.06 ± 41.75	105.16 ± 49.66	0.98*
Ischemic time (min.) (Mean ± SD)	72.90 ± 27.52	71.65 ± 32.16	0.72*
Ultrafiltration (mL) (Mean ± SD)	1877.43 ± 1135.78	1838.27 ± 853.01	0.75*
Total external electric socks (N), Median (min-max)	2 (0-5)	1 (0-5)	0.97**
EF preoperative (%) (Mean ± SD)	50.94 ± 8.43	54.23 ± 6.83	0.0009**
EF postoperative (%) (Mean ± SD)	49.85 ± 7.83	51.99 ± 5.92	0.0166**

A statistically significant difference is observed when comparing the mean values of preoperative and postoperative hs-cTnT, CK-MB, lactate, and seven-day postoperative EF, as demonstrated in Table 3.

**Table 3:** Perioperative blood tests and ejection fraction. Mann-Whitney test; *P* < 0.05 statistically significant. hs-cTnT, high-sensitivity troponin I; CK-MB, creatine kinase MB

Variables		Male (*n* = 190)	*P*-value	Female (*n* = 110)	*P*-value
hs-cTnT (ng/L) (Mean ± SD)	Preoperative	0.83 ± 0.59	0.0001	0.90 ± 0.69	0.0001
	Postoperative	1.16 ± 0.70		1.24 ± 0.77	
CK-MB (ng/L) (Mean ± SD)	Preoperative	24.02 ± 7.88	0.0001	23.45 ± 9.19	0.0001
	Postoperative	33.17 ± 11.04		32.74 ± 9.29	
Lactate (mmol/L) (Mean ± SD)	Preoperative	1.30 ± 0.50	0.0001	1.37 ± 0.55	0.001
	Postoperative	1.57 ± 0.69		1.68 ± 0.70	
pH (Mean ± SD)	Preoperative	7.19 ± 0.39	0.52	7.15 ± 0.36	0.01
	Postoperative	7.22 ± 0.41		7.29 ± 0.45	
LV ejection fraction (%) (Mean ± SD)	Preoperative	50.95 ± 8.46	0.05	54.23 ± 6.83	0.0001
	Postoperative	49.85 ± 7.87		51.99 ± 5.92	

A decrease in contractility was observed using transthoracic echocardiography in female and male subjects 30 days after surgery, with a mean decrease of 1.1% and 2.2%, respectively (*P* < 0.001).

The postoperative evolution regarding diuresis, ACU stay, inotropic support, and intubation time were similar between the groups. The mortality rate was 6 (5.45%) in the female group compared to 9 (4.73%) in the male cohort (*P* = 0.53). From an arrhythmic perspective, tachyarrhythmias were more prevalent in the female cohort, the observed differences were statistically significant. The male group exhibited a higher incidence of benign ventricular arrhythmias, specifically isolated ventricular extrasystoles (1.49% more, *P *= 0.72). Additionally, the male group had a higher prevalence of re-exploration for bleeding (5.78% vs. 3.63%, *P *= 0.58). These findings are illustrated in Table 4.

**Table 4:** Postoperative evolution and adverse events. *Mann-Whitney test. **Chi-square test. *P* < 0.05 statistically significant. ACU, Acute Care Unit

Variables	Male (*n* = 190)	Female (*n* = 110)	*P*-value
Diuresis (mL/day) (Mean ± SD)	2051.74 ± 882.49	2086 ± 998.14	0.75*
ACU stay (days), Median (min-max)	2 (1-15)	2 (1-12)	0.69*
Inotropic support (days), Median (min-max)	1 (0-15)	1 (0-9)	0.64*
Intubation (hours), Median (min-max)	9 (4-85)	9 (3-136)	0.34*
Re-exploration for bleeding (%)	5.78 (*n* = 11)	3.63 (*n* = 4)	0.58**
Atrial fibrillation (%)	7.36 (*n* = 14)	10 (*n* = 11)	0.54**
Ventricular fibrillation (%)	3.15 (*n* = 6)	4.54 (*n* = 5)	0.75**
Ventricular tachycardia (%)	2.10 (*n* = 4)	3.63 (*n* = 4)	0.67**
Isolated ventricular extrasystoles (%)	4.21 (*n* = 8)	2.72 (*n* = 3)	0.72**
Neurological disfunction (%)	2.10 (*n* = 4)	2.72 (*n* = 3)	0.64**
Mortality (%)	4.73 (*n* = 9)	5.45 (*n* = 6)	0.53**

Table 5 presents the regression model for postoperative LVEF and the associated covariates.

**Table 5:** Regression model for postoperative LV ejection fraction and covariates. *P* < 0.05 statistically significant. LV, left ventricle; LVEF, left ventricular ejection fraction; CI, confidence interval

Variables	Postoperative LVEF				
	*P*-value		Odds ratio	95% CI	
Age	0.51	0.5769			0.2389-1.3931
Female	0.50	2.0199			0.7408-5.5076
Preoperative LVEF (%)	0.0001	0.0705			0.0305-0.1633
Total intervention time (minutes)	0.61	1.4967			0.6551-3.4195
Bypass time (minutes)	0.70	3.7327			0.6066-22.9698
Ischemic time (minutes)	0.50	0.2817			0.0368-2.1535

In the multivariate logistic regression model (Table 6), the dependent variable (seven-day mortality) was found to be influenced exclusively by ischemic time (odds ratio [OR] = 1.034, 95% CI = 1.016-1.052, *P *= 0.0001). Furthermore, we identified statistically significant co-variates for 30-day mortality, namely preoperative LVEF (OR = 0.746, CI = 0.598-0.930, *P *= 0.009), postoperative LVEF (OR = 1. 234, 95% CI = 1.031-1.477, *P* = 0.02), ischemic time (OR = 1.027, 95% CI = 1-1.054, *P *= 0.04) and duration of inotropic support (OR = 1.588, 95% CI = 1.056-2.389, *P* = 0.02).

**Table 6:** Regression model for 7- and 30-day mortality and covariates. *P* < 0.05 statistically significant. LVEF, left ventricular ejection fraction; CI, confidence interval; OR, odds ratio

Variables	7-day mortality			30-day mortality		
	*P*-value	OR	95% CI	*P*-value	OR	95% CI
Age	0.08	3.452	0.842-14.159	0.31	0.169	0.005-5.223
Gender	0.22	0.428	0.110-1.666	0.84	1.356	0.066-27.830
Preoperative LVEF	0.53	0.969	0.877-1.070	0.009	0.746	0.598-0.930
Postoperative LVEF	0.89	0.993	0.892-1.105	0.02	1.234	1.031-1.477
Ischemic time (minute)	0.000	1.034	1.016-1.052	0.04	1.027	1.000-1.054
Inotropic support duration (days)	0.63	1.081	0.787-1.485	0.02	1.588	1.056-2.389

## Discussion

In the field of cardiac pathology, to achieve surgical excellence, the surgeon requires a bloodless field and electromechanical quiescence [7]. These conditions are detrimental to achieving cardioprotection and technical accuracy. Today, several cardioplegic agents have proven to be safe and practical when implemented in the cardioprotective algorithm. The ideal cardioplegic solution is not yet known and debates on this topic continue [8,9]. Currently, the field of cardiac surgery is under increasing pressure due to the growing aging population requiring surgical intervention.

 The gender distribution of patients presenting with aortic pathology and requiring surgical intervention in the present study aligns with international demographic data. Our findings also indicate that in our study, males are more likely to develop aortic valve pathology (*n* = 190, 62.9%) than females (*n *= 110, 37%). The diagnosis of aortic valvulopathy is more probable in the sixth decade of life in both genders.

From database records, we retrieved certain perioperative elements that, in general understanding, can influence postoperative evolution. To the best of our knowledge, no research to date has been able to quantify the impact of these perioperative records according to gender in the case of aortic valve replacement using Bretschneider cardioplegia.

In our study, it was observed that the mortality rate at 30 days was 5.45% (*n* = 6) among female patients and 4.73% (*n *= 9) among male patients. The analysis revealed that gender was not an independent predictor of early mortality. In our analysis, the patient was monitored throughout their hospitalization, or for a period of up to 30 days following surgery. Consequently, it is not possible to calculate long-term mortality. Other studies have submitted comparable results, indicating no statistically significant difference in postoperative mortality between the genders [10]. Overall, the postoperative survival gap between genders has narrowed in recent decades due to the development of more sophisticated diagnostic tools and the implementation of well-established surgical protocols for each patient. In general, gender has not been a primary focus of major analyses concerning survival rates after cardiac surgery. This is due to the larger number of male patients, which results in a reduction in statistical power. However, the considerable heterogeneity of the elements involved in the diagnosis and management of aortic pathology results in markedly different findings in the reports of surgical centers. In contrast, other studies have reported higher mortality rates for women [11,12]. The following factors have been identified as contributing to an increased mortality rate in women compared to men: a higher body mass index, an increased number of comorbidities, advanced age, more technically demanding procedures (e.g., smaller aortic annulus), and late diagnosis of aortic pathology [13].

From the perspective of surgical intervention, the division of patients by gender and subsequent comparison of results reveals a similarity between the ischemic and bypass times for these two groups. Table 2 indicates a tendency for a prolonged total intervention time in the female group; however, this tendency did not reach statistical significance. As previously demonstrated by various studies, a prolonged surgical procedure will inevitably influence the postoperative evolution due to prolonged tissue trauma, inflammatory response, fibrinolysis, and other factors, which are a direct cause of an increased incidence of adverse events such as postoperative rhythm disorders, neurological dysfunction, renal impairment, and other [14,15].

Myocardial protection was quantified by analyzing the EF, cardiac enzymes, and the incidence of rhythm disturbances. A comparison of the median EF between groups (one day before and seven days after surgery) demonstrated that the decline was more pronounced in males than in females. In the preoperative period, women with aortic valve disease demonstrate greater capacity to preserve EF than men. However, postoperatively, there is a notable reduction in EF in women relative to men, yet seven days after surgery, women's values still exceed those of men. One potential explanation for this discrepancy is that hormonal factors are involved. Research has demonstrated that hormonal action plays a role in maintaining myocyte number in women and reducing the number of cardiac cells in men over time, due to apoptosis. This phenomenon is compensated for by the formation of fibrosis and an increase in the extracellular matrix. Consequently, women possess a contractile reserve that enables them to maintain EF, both preoperatively and postoperatively (Tables 2-3) [16].

Although there was an observable increase in the incidence of postoperative atrial fibrillation in women, this did not reach statistical significance. The majority of studies identify older age as the primary risk factor, rather than gender. The odds ratio for developing postoperative atrial fibrillation is 1.51 per decade. In the same studies, the incidence of postoperative atrial fibrillation is presented as being much higher (between 37 and 50%) than that observed in our study [17]. The use of Bretschneider cardioplegia within our study group may be a potential cause for these differences, as previous studies have demonstrated a protective effect against postoperative arrhythmias [18].

The analysis of hs-cTnT and CK-MB dynamics demonstrated an immediate postoperative increase in values, with no significant intergroup differences observed. The damage to cardiac cells that occurs following AVR is a multifactorial process that encompasses several factors. These include the efficacy of the cardioplegic agent, iatrogenic coronary ostial injury, cardiac manipulation, the cooling-rewarming process of the heart, the patient's basal cardiac status, defibrillation, and the development of ischemia/reperfusion lesions. It is anticipated that patients undergoing on-pump cardiac interventions will demonstrate a degree of cardiac enzyme release, with troponin exhibiting a higher degree of specificity than sensitivity. It is reasonable for clinicians to consider further investigation if cardiac enzyme levels are higher than anticipated. The current diagnostic protocols for new-onset cardiac ischemia specify a range of cardiac enzyme levels that are 10 to 70 times higher than baseline levels, which is considerably higher than the upper limit of the test kits [19,20]. The results of our study did not yield any values that were as high as those previously reported in the literature. This can be attributed to the nature of the pathology, which does not involve epicardial coronary arteries. In cases of acute coronary events, values that are several times higher than the maximum normal value are most often observed. Conversely, it has been demonstrated that there is robust protection of hypertrophic myocardium when crystalloid, cold Bretschneider solution is utilized. The rheology and the considerable quantity of buffer solutions present within HTK cardioplegia make this pathology an optimal candidate for the deployment of this particular type of cardioplegia, in alignment with the findings of previous studies [7,21,22]. The results of preceding research have demonstrated that there is a strong causal relationship between a notable elevation in cardiac enzyme levels within the initial 24 hours following cardiac surgery and in-hospital, medium, and long-term mortality, even in the absence of post-procedural myocardial infarction (type 5 MI) [23].

It is well documented that neuropsychiatric deterioration is a further adverse event that may occur following cardiac surgical interventions, particularly those involving cardiopulmonary bypass. Postoperative neurocognitive impairment may be the result of a subsequent inflammatory response, specifically neuroinflammation. From the standpoint of neurological inquiry, our findings indicate that there is no evidence to suggest that gender exerts an influence in the immediate postoperative period. These findings are those previously documented in the literature [24]. A study published in 2019 of 2121 patients who underwent cardiac surgery between 2008 and 2013 found that the incidence of major stroke was 1.7%, with 2.5% experiencing transient ischemic attacks (including delirium, psychosis, and convulsions). The mean age was 65.3 ± 12.1 years, with 60 (65.9%) of the patients being male [25]. A review of the literature reveals that aortic valve surgery has the highest incidence of neurological complications compared to other surgical procedures [26]. Although transcatheter aortic valve implantation (TAVI) procedures have long been known to be linked with an elevated risk of adverse neurological events, with incidence rates exceeding those of traditional surgery, recent studies have revealed an inversion of this relationship [27]. Aortic valve surgery represents the procedure with the greatest risk of either silent or manifest neurological complications. The neurocognitive status of patients following aortic valve replacement is influenced by several additional factors, classified as either changeable (perioperative-related) or non-changeable (patient status and history). The first category of factors is largely predictable and includes surgical approach (median full/partial sternotomy), embolic load, hemodynamic variations during cardiopulmonary bypass, cannulation site, core temperature, anesthesia management, inflammatory status, glycemic control, and others [28,29]. Baseline patient characteristics, including biological age, tissue fragility, and preoperative history of alcoholism, depression, or other neuropsychiatric symptoms, are of particular concern as they are non-modifiable and can have a significant impact on postoperative outcomes [30-32]. The occurrence of neurocognitive impairment in the postoperative period is a factor for increased mortality and long-term neuropsychic deterioration, with 30% of patients at risk of developing dementia [33,34].

Postoperative hemorrhages are a major cause of death after cardiac surgery [35]. Our analysis indicates an increasing trend towards surgical reintervention for hemostasis, with a higher incidence in males. However, this trend did not reach statistical significance. Previous studies have identified male gender as a known patient-related predictor for excessive postoperative bleeding [36,37] Furthermore, research has demonstrated that women exhibit augmented clot strength and accelerated fibrin formation as men [38]. A multitude of factors influence postoperative chest drainage (such as the patient's coagulation factor function, duration of cardiopulmonary bypass, anesthetics, and procoagulants used intraoperatively) [39]. The contribution of the surgical team is of paramount importance, as it can significantly reduce postoperative bleeding through the rigorous implementation of a personalized check protocol before chest closure. In surgical cases involving aortic valve pathology, especially aortic stenosis, hemostasis is a particular concern. There are several competing aspects, including acquired coagulopathy and induced vasodilatation. In the first case, the existing high share rheological stress induces proteolysis of von Willebrand factor multimers (resulting in a component of the Heyde syndrome), impaired platelet adhesion and aggregation, hypofibrinolysis [40,41]. The second condition is caused by the physiopathology of aortic stenosis, precisely microvascular vasodilatation secondary to humoral autoregulation. Consequently, diffuse bleeding may frequently occur in all layers of surgical dissection, which is often refractory to local hemostatic maneuvers [42,43].

Table 5 illustrates that preoperatory LVEF significantly influences postoperative LVEF. This conclusion can also be logically deduced; however, the element that stands out is the postoperative LVEF independence from the other analyzed parameters. There is a great debate regarding the effect of surgical times, including ischemic, bypass, and total intervention times, on postoperative LVEF. Some surgeons promote shortening these times due to secondary negative effects on the myocardium and other organs. They emphasize expediency over meticulousness in every surgical maneuver. In contrast, other surgeons prioritize surgical precision, which often results in a longer ischemic time. The results of our study provide further evidence in support of the second opinion. Nevertheless, ischemic time exerts a significant influence on the early postoperative state, as it can influence postoperative seven-day mortality, as illustrated in Table 6. In the multivariate logistic regression model, the dependent variables, specifically seven-day postoperative mortality, are influenced exclusively by aortic cross-clamping duration. Corroborating this finding with the aforementioned results we can conclude that ischemic time impacts postoperative results not via a direct myocardial depression, but as being a marker of complex interventions with prolonged duration for correction. This observation provides evidence that the Bretschneider solution can effectively protect the myocardium in complex cases. [21].

The preoperative LVEF is also the primary covariate that determines the 30-day postoperative mortality rate. This is evidenced by the fact that a low ejection fraction is associated with a decreased postoperative survival rate. Halkos et al. identified EF as a determinant of postoperative mortality in a univariable analysis of 773 patients who underwent aortic valve replacement [44]. A study carried out at the Mayo Clinic in Rochester, MN, on 2017 patients undergoing aortic valve replacement revealed the association between preoperative EF and survival. A reduction in postoperative survival was observed even in patients with an EF between 50% and 59% when compared to those with an EF > 60%. This increase in mortality, also in patients with an EF above the classical threshold for cardiomyopathy, is attributable to the alteration of the longitudinal function of the EF, which precedes the dysfunction of the concentric function [45]. A critical period exists, commencing with the onset of symptoms and extending until the ejection fraction begins to decline. If the surgical procedure is performed within this critical timeframe, the patient will most benefit, with a reduction in complications and an improved survival rate. These findings contribute to an ongoing debate regarding the optimal timing for surgical intervention, despite the existence of various international protocols. Some institutions adopt a more liberal approach. [46-48].

Study limitations

As this study employs a retrospective design, the data have inherent limitations, including potential biases introduced by patient selection and cohort heterogeneity. A further limitation of the study is that the results may be subject to influence from factors not accounted for, such as discrepancies in the perioperative monitoring and care of patients. It is not possible to apply an objective method of quantifying surgical technique. Consequently, any study that includes surgical patients will be subject to this limitation. Further studies analyzing postoperative evolution should include a larger patient population to mitigate the impact of these limitations.

## Conclusions

The use of HTK cardioplegia in aortic valve surgery yields comparable outcomes, effectively overcoming the disparate structural and microcirculatory alterations between genders. The impact on postoperative evolution is variable, with the cardioplegic solution emerging as a significant contributing factor. Further investigation into patients with multiple comorbidities, such as impaired cardiac function, is necessary to elucidate the potential influence of these factors on the susceptibility of the myocardium to ischemia-reperfusion injury. It can be stated that Bretschneider cardioplegia is a safe and effective method of myocardial protection for adult patients undergoing AVR.

Our study has enabled us to ascertain certain findings that highlight potential discrepancies in the perioperative management of patients based on individual characteristics. Understanding these differences and their underlying mechanisms may facilitate the improvement of outcomes. To the best of our knowledge, this study is the only one to highlight the pitfalls of crystalloid cardioplegia infusion, particularly concerning the morphopathological changes in the LV secondary to aortic valvulopathy. Moreover, it has also drawn attention to the differing characteristics of these changes between sexes. 
